# Combination Immunotherapy Using Oncolytic Virus for the Treatment of Advanced Solid Tumors

**DOI:** 10.3390/ijms21207743

**Published:** 2020-10-19

**Authors:** Chang-Myung Oh, Hong Jae Chon, Chan Kim

**Affiliations:** 1 Department of Biomedical Science and Engineering, Gwangju Institute of Science and Technology (GIST), Gwangju 61005, Korea; cmoh@gist.ac.kr; 2 Medical Oncology, CHA Bundang Medical Center, CHA University School of Medicine, Seongnam 13497, Korea

**Keywords:** oncolytic virus, combination immunotherapy, immune checkpoint inhibitor, tumor microenvironment

## Abstract

Oncolytic virus (OV) is a new therapeutic strategy for cancer treatment. OVs can selectively infect and destroy cancer cells, and therefore act as an in situ cancer vaccine by releasing tumor-specific antigens. Moreover, they can remodel the tumor microenvironment toward a T cell-inflamed phenotype by stimulating widespread host immune responses against the tumor. Recent evidence suggests several possible applications of OVs against cancer, especially in combination with immune checkpoint inhibitors. In this review, we describe the molecular mechanisms of oncolytic virotherapy and OV-induced immune responses, provide a brief summary of recent preclinical and clinical updates on this rapidly evolving field, and discuss a combinational strategy that is able to overcome the limitations of OV-based monotherapy.

## 1. Introduction

Viruses are continuously co-evolving with our immune systems and have developed sophisticated mechanisms to manipulate host immune responses [1]. With scientific advances in the understanding of the molecular interplay between viruses and host immune systems, we can develop a new therapeutic modality against cancers using finely tuned viruses, called viroceuticals [2,3]. These “oncolytic” viruses (OVs) can selectively infect and kill cancer cells while sparing normal cells [4,5,6]. Some viruses such as myxoma virus or reovirus have inherent selectivity to tumor cells, while being nonpathogenic in healthy human cells [7]. On the other hand, other OVs, including adenovirus, herpes simplex virus type-1 (HSV-1), and vesicular stomatitis virus (VSV), have been genetically engineered to function as vectors to boost anti-tumor immune responses [8]. The anti-tumor efficacies of these OVs have been evaluated in many preclinical and clinical studies as monotherapy and combination therapy [2,5,9,10,11,12,13,14,15]. In 2015, oncolytic herpesvirus, talimogene laherparepvec (Imlygic, also known as T-VEC), was granted U.S. Food and Drug Administration (FDA) and European Medicine Agency (EMA) approval and is now being used for the treatment of patients with advanced melanoma [16,17].

A big breakthrough has been achieved in the field of cancer immunotherapy over the last decade. Immune checkpoint inhibitors (ICIs) targeting cytotoxic T lymphocyte-associated protein 4 (CTLA-4); programmed cell death 1 (PD-1); and PD-1’s main ligand PD-L1, have been introduced for the treatment of more than a dozen types of cancers [18,19,20]. However, only 20–30% of patients respond to ICI monotherapy, while others show intrinsic resistance to ICI treatment due to a non-inflamed cold tumor microenvironment (TME) [8,21,22]. These non-inflamed cold tumors are also described as “immune deserts” because they are poorly immunogenic and have very few anti-tumor immune effector cells within the TME [5,23,24]. Therefore, there have been tremendous efforts to develop a novel immunotherapeutic agent that can not only enhance tumor immunogenicity but also augment immune cell trafficking into the TME to covert non-inflamed cold tumors to inflamed hot tumors that can respond favorably to ICI therapy.

In this review, we will summarize OVs’ mode of action and provide recent preclinical and clinical evidence supporting OV as an ideal platform for combination immunotherapy.

## 2. Mechanism of OVs’ Anti-Tumor Effects

Tumor selectivity is an essential prerequisite of OVs to guarantee maximal oncolysis while minimizing off-target effects on normal tissue [2]. Since the 1990s, with the development of molecular virology, the genomes of wild-type viruses have been engineered to enhance their tumor selectivity. There are several ways to enhance the tumor selectivity of OVs. Because tumor cells activate various oncogenic signaling pathways during carcinogenesis, engineering viruses that depend on these oncogenic pathways can remarkably increase their tumor selectivity without affecting normal tissues [4,8,25]. For example, the oncolytic vaccinia virus, pexastimogene devacirepvec (Pexa-Vec), was engineered to inactivate its own thymidine kinase (TK) gene for tumor selectivity. Because TK is essential for nucleic acid metabolism, Pexa-Vec can preferentially replicate within TK-overexpressing cancer cells, while not being able to do it within normal healthy cells where TK activity is absent or minimal, thus showing tumor selectivity [5,14].

**Figure 1:**
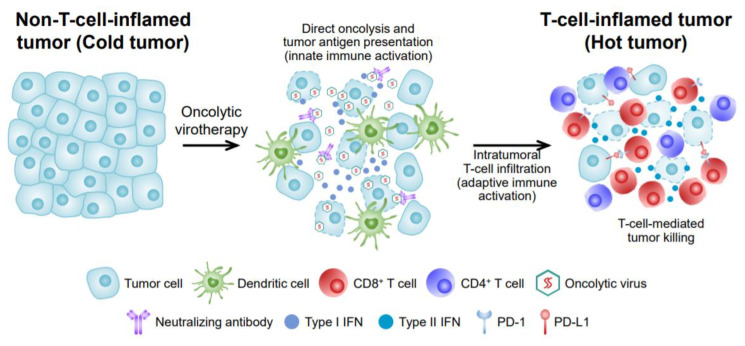
Mechanisms of oncolytic virus (OV) anti-tumor effects.

The therapeutic efficacy of OVs depends on two main modes of action (Figure 1). (1) OVs can inhibit protein synthesis of tumor cells and destroy infected tumor cells by self-replication. After viral infection, OVs continue self-replication until the cell bursts. (2) OVs can recruit and activate tumor-infiltrating immune cells by releasing a large amount of tumor antigens and secreting cytokines [2,8,22]. When an OV directly breaks tumor cells, viral antigens, tumor antigens, and damage-associated molecular patterns are massively released from dying tumor cells. Therefore, OV can be used as an in situ antigen-agnostic cancer vaccine within the TME [2,5,24]. This can rapidly trigger acute innate immune responses consisting of dendritic cells, macrophages, and natural killer (NK) cells. These cells can further destroy OV-infected tumor cells and secrete pro-inflammatory cytokines. Moreover, these innate immune cells uptake viral and tumor antigens and present them for T cell activation. Finally, activated T cells proliferate and accumulate within the TME and exert their effector functions against cancer cells [2,8,24,25,26,27]. In particular, this T cell-mediated adaptive immunity plays a critical role in durable cancer control after oncolytic virotherapy and enables effective control of tumor cells at distant sites beyond the locoregional site of OV injection [2,24]. Overall, OV can remodel non-inflamed cold tumor to T cell-inflamed hot tumors by enhancing tumor immunogenicity and augmenting intratumoral T cell infiltration.

Besides tumor cells and immune cells, other cellular components within the TME also respond to OV therapy [5,28,29,30]. Many OVs can infect and destroy tumor endothelial cells, thus showing direct vascular disruption. This anti-angiogenic effect was selective for tumor endothelial cells but not for endothelial cells of normal tissues, indicating the targeted destruction of pathologic tumor vasculatures [28,29]. Arulanandam et al. suggested a potential mechanism for this viral tropism of tumor blood vessels. In their study, activated vascular endothelial growth factor receptor 2 (VEGFR2) signaling within tumor endothelial cells upregulated the transcriptional repressor, positive regulatory domain I–binding factor 1 (PRD-BF1), which suppresses genes involved in type I interferon-mediated anti-viral activity, thereby making tumor vessels sensitive to OV infection [30]. Cancer-associated fibroblasts (CAFs) also respond to OV therapy. Although normal fibroblasts are refractory to OV infection, CAFs have increased sensitivity to OV therapy. Tumor cell-derived transforming growth factor beta (TGF-β)-reprogrammed CAFs suppress their innate anti-viral and type I interferon signaling, thereby rendering CAFs sensitive to OV infection. In turn, CAFs dampen the anti-viral response within tumor cells by secreting high levels of fibroblast growth factor 2 (FGF2). Therefore, cellular crosstalk between CAFs and tumor cells promotes OV growth and killing in both cell types [31].

## 3. OV Monotherapy

Over the past two decades, OV therapeutics have grown very rapidly with the advancement of molecular biology, virology, immunology, and genetic engineering [32]. Table 1 summarizes the OVs currently in development and their associated genetic modifications [33].

### 3.1. Melanoma

Melanoma is one of the most sensitive types of malignancy for cancer immunotherapy. T-VEC demonstrated its clinical efficacy in patients with melanoma and has inaugurated the era of oncolytic virotherapy. T-VEC is the first FDA-approved oncolytic herpesvirus, genetically modified to selectively replicate within tumor cells and to increase tumor antigen presentation by dendritic cells through granulocyte-macrophage colony-stimulating factor (GM-CSF) transgene expression [16]. HSV-1 is a double-stranded DNA virus, inherently highly lytic, which can infect skin and peripheral nerves, thereby causing recurrent fever blisters such as skin vesicles or mucosal ulcers under high-stress conditions [52]. T-VEC has been engineered to avoid the development of fever blisters by deleting the neurovirulence gene, infected cell protein 34.5 (ICP34.5). Moreover, it uses surface nectins to selectively penetrate tumor cells and proliferate within by using disrupted oncogenic and anti-viral pathways such as protein kinase R (PKR) and type I interferon (IFN) pathways [53].

OPTiM (ClinicalTrials.gov NCT00769704) was a randomized phase III trial that compared T-VEC and GM-CSF in patients who had histologically confirmed unresectable stage IIIB/C/IV melanoma with at least one cutaneous, subcutaneous, or nodal lesion [13,54]. By conclusion, OPTiM reported that T-VEC improved longer-term efficacy versus GM-CSF alone. T-VEC also showed durable complete responses (CR), resulting in long-term overall survival. The medial overall survival (OS) was 23.3 months (95% confidence interval (CI): 19.5–29.6) and 18.9 months (95% CI: 16.0–23.7) in the T-VEC and GM-CSF arm, respectively [34]. Although T-VEC is the only OV approved by the FDA for patients with melanoma, further clinical trials using other OVs such as HF10 (canerpaturev), coxsakievirus, pelareorep, and vaccinia-GM-CSF are currently ongoing in patients with melanoma [33].

### 3.2. OVs against Other Malignant Cancers

The anti-tumor effects of OVs have been investigated in other malignancies as well. Malignant gliomas are highly aggressive primary brain tumors. Three oncolytic HSV-1 strains (HSV1716, G207, and G47∆) have completed phase I trials in glioma patients, and phase II trials with these OVs are now ongoing [33]. The third generation HSV-1, G47∆, have shown significant anti-tumor effects in a phase II clinical trial in patients with glioblastoma. When G47∆ was stereotactically injected into patients with recurrent or residual glioblastoma in addition to maintenance chemotherapy with temozolomide, the 1-year survival rate was 92.3%. Because statistical significance was higher than the criteria of early termination, the study was terminated early, and a further pivotal trial is now under development [55]. Oncolytic adenoviruses such as ONY015 (previously known as AD2/5 dl1520) and DNX-2401(also known as tasadenoturev) are also being evaluated in phase I or phase II clinical trials [33].

Pancreatic cancer remains one of those with the poorest prognosis and major causes of cancer-related deaths worldwide [56]. Its poor prognosis is related to its unique TME, which is poorly immunogenic with very low expression of tumor neoantigens, thus limiting anti-cancer immune responses. Even worse, the TME of pancreatic cancer is enriched with immunosuppressive stromal cells and has a very dense fibrotic extracellular matrix (ECM), which acts as a biophysical barrier disturbing intratumoral delivery of anti-cancer drugs and immune cells. These factors suppress the anti-tumor effects of both chemotherapy and immunotherapy in patients with pancreatic cancer [57,58,59]. To overcome these unfavorable TMEs in pancreatic cancer, several OVs have been investigated in preclinical in vivo models, and some OVs are already under development in phase I and II clinical trials. An oncolytic adenovirus, OBP-702, expressing tumor suppressor p53, significantly suppressed tumor growth in an orthotropic xenograft model of pancreatic cancer by disruption of extracellular signal regulated kinase (ERK) signaling [60]. Oncolytic adenovirus (ONYX-015, VCN-01, LOAd703), oncolytic HSV (T-VEC, HF10, Orien X010), and pelareorep (Reolysin) are now in phase I or phase II clinical trials [33].

Breast cancer is the most commonly diagnosed cancer among women. Despite recent advances in molecularly targeted therapies, there are still treatment-resistant cases; thus, OVs have become new therapeutic options against these intractable breast cancers. Various types of OVs have been investigated in patients with breast cancer. The novel oncolytic HSV-encoding interleukin 12 (designated G47Δ-mIL12) showed significant anti-tumor effects in a preclinical model of syngeneic triple-negative breast cancer (4T1) [61]. Oncolytic HSV (T-VEC, HF10), adenovirus (ONYX-015), vaccinia virus (VVDD), Newcastle disease virus (PV701), and pelareorep (Reolysin) are in phase I or phase II clinical trials [33].

## 4. OV Combination Immunotherapy

Because OVs as single agents showed limited efficacy in clinical trials [33], a combination of OVs with other anti-cancer agents is being tested to overcome this limitation. Among various agents, the combination of OV with immunotherapeutic agents, especially ICI, has been extensively studied because OV is a natural activator of both innate and adaptive immunity.

### 4.1. Combination Therapy with OVs and ICIs

Immune checkpoint blockade has revolutionized the therapeutic landscape of advanced cancer over the last decade. ICIs are monoclonal antibodies that block immune checkpoint proteins, such as PD-1, PD-L1, or CTLA-4, which are natural brakes of the immune system, from interacting with their binding partners [19,62]. This interrupts the immunologic shutdown signal being sent to T cells so that they can recognize and attack tumor cells [8,15]. Since 2011, the FDA approved six ICIs—ipilimumab (anti-CTLA-4), nivolumab (anti-PD-1), pembrolizumab (anti-PD-1), atezolizumab (anti-PD-L1), avelumab (anti-PD-L1), and cemiplimab (anti-PD-1)—for the treatment of more than 15 different types of cancer [63]. However, the overall response rate to ICI monotherapy remains approximately 20 to 30% because a significant number of tumors lack tumor antigens for T cell-priming (low immunogenicity) and have little effector T cell infiltration within the TME [19,22,23]. OV can increase tumor immunogenicity by acting as an in situ cancer vaccine and promote intratumoral T cell infiltration, serving as an ideal immunologic platform to potentiate and expand the anti-tumor efficacy of ICIs [5,24].

Recently, the combination of OVs with ICIs has been intensively investigated in many clinical trials (Table 2). Combinations of ICIs with either unmodified or modified OVs armed with cytokines and chemokines have demonstrated promising therapeutic efficacies for metastatic or unresectable tumors [6,64,65]. T-VEC is leading this promising combination immunotherapy. In a recent phase II trial for patients with stage IIIB to IV melanoma, 198 patients were 1:1 randomized to T-VEC in combination with ipilimumab or ipilimumab (ipi) alone. The combo arm showed a significantly higher objective response rate compared to ipi monotherapy (36.7% vs. 16.0%, *p* = 0.022). The median progression-free survival was 13.5 months with combo therapy and 4.5 months with ipi monotherapy. Therefore, the T-VEC plus ipi combo showed remarkable tumor burden reduction and durable activity in patients with melanoma [66].

In a phase Ib trial for patients with advanced melanoma, 21 patients were treated with T-VEC followed by combination therapy with pembrolizumab. This combination therapy was well tolerated and showed no dose-limiting toxicities. The objective response rate and complete response rate were 62% and 33%, respectively. Patients who responded to combination therapy demonstrated increased intratumoral cluster of differentitiation 8^+^ (CD8^+^) T cells, elevated PD-L1 expression, and IFN-γ gene expression after T-VEC treatment. However, objective responses were not associated with baseline CD8^+^ T cell infiltration or baseline IFN-γ signature. These results indicate that T-VEC may improve the efficacy of pembrolizumab by changing the TME [65]. On the basis of these promising findings, an ongoing phase III trial is currently comparing systemic administration of pembrolizumab with intralesional injection of T-VEC or placebo in patients with stage IIIB-IV melanoma.

On the basis of tolerable safety for intrahepatic injection of T-VEC [67], a phase Ib trial of intrahepatic T-VEC injection in combination with intravenous pembrolizumab assessed the maximum tolerated concentration (MTC) in patients with progressive hepatocellular carcinoma (HCC), breast cancer, colorectal cancer, gastroesophageal cancer, melanoma, non-small cell lung cancer, or renal cell cancer with liver metastasis and reported MTC and tolerability in an American Society of Clinical Oncology (ASCO) 2020 meeting [68]. MTC was 108 plaque forming units (PFU)/mL in non-HCC patients, and exploration of MTC in the HCC population is ongoing. There were no fatal adverse events. Thus, intrahepatic injection of T-VEC in combination with intravenous anti-PD-1 therapy has demonstrated feasibility and tolerability.

The ONCOS-102 is a chimeric oncolytic adenovirus armed with human GM-CSF and Ad5/3 chimeric capsids, and it has shown promising anti-tumor effects through the upregulation of PD-L1 in a phase I clinical trial against solid tumors (NCT01598129) [10]. Thus, ONCOS-102, in combination with ICIs, are being tested for advanced solid malignancies such as prostate cancer, melanoma, and peritoneal cancers in clinical trials (Table 1 and Table 2).

In a phase Ib trial for patients with metastatic or unresectable renal cell carcinoma (REN026), IV infusion of Pexa-Vec also reported promising results in combination with cemiplimumab (anti-PD-1) in an American Association for Cancer Research (AACR) 2020 meeting [69]. The overall response and disease control rates in 16 evaluable patients were 37.5% (one complete response and five partial responses) and 75%, respectively. Overall, 12 out of 210 adverse events (5.7%) were reported as grade 3, which includes fever, flu-like symptoms, blood pressure changes after Pexa-vec infusion, and pneumonia, which are mostly transient. Thus, IV Pexa-Vec and cemiplimab combination therapy showed an acceptable safety profile in patients with renal cell carcinoma. Further investigation is ongoing with an expansion cohort and another cohort with intratumoral Pexa-Vec and cemiplimab combination therapy.

### 4.2. Combination Therapy with OVs and CAR-T Therapy

Chimeric antigen receptor T (CAR-T) cell therapy implies in vitro design, modification, and amplification of T cells from a patient’s blood to grant them the ability to recognize the surface antigens on tumor cells via the transduced CAR structure on the T cell surface. After amplification in the laboratory, these CAR-T cells are administered intravenously to the patient with cancer. In a mouse neuroblastoma model, oncolytic adenovirus armed with the chemokine RANTES (Regulated upon Activation, Normal T Cell Expressed and Presumably Secreted) and the cytokine interleukin-15 (Ad5Δ24) enhanced migration and proliferation of CAR-T cells [70]. Therefore, the combination therapy of Ad5Δ24 and CAR-T cells significantly increased the overall survival of tumor-bearing mice [70]. The combination of HER2/neu (also known as ErbB-2) CAR-T cells and oncolytic adenovirus-expressing anti-PD-L1 and IL-2 remarkably improved survival compared with either form of therapy alone in head and neck squamous cell carcinoma xenograft mice [71]. Moreover, Park et al. reported a novel therapeutic combination using both OVs and CAR-T therapy in their recent study [72]. They used an oncolytic chimeric orthopoxvirus carrying CD19t (OV19t) to generate CD19t at the tumor cell surface. After the administration of the OV19t and CD19-CAR T cell combination, ~70% of tumor cells were positive for CD19t and showed increased CAR-T cell infiltration in human tumor (MDA-MB-468) xenograft mice [72]. These preclinical studies suggest the promising potential of this combination therapeutic strategy for cancer treatment.

### 4.3. Combination Therapy with OVs and BiTEs

Bispecific antibodies are a novel class of anti-tumor agents that simultaneously target two different types of antigens or epitopes by combining two antibodies [73]. Bispecific T cell engagers (BiTEs) are a subclass of bispecific antibodies that have specific antibodies for CD3 on one arm and another specific antibody for a tumor antigen on the second arm [74]. Blinatumomab, a well-characterized BiTE targeting both CD19 and CD3 was approved by the FDA for the treatment of a rare type of acute lymphoblastic leukemia (ALL) in 2017 [74]. Catumaxomab (Removab) and MT110, both targeting epithelial cell adhesion molecule (EpCAM) and CD3, were investigated in various cancers such as ovarian and gastric cancer in phase II or III clinical trials [75]. Ertumaxomab and HER2Bi-aATC, both targeting HER2 and CD3, were tested for their anti-tumor efficacy against breast cancer in phase I/II clinical trials [75].

BiTE applications in solid tumors have shown some limitations, such as low tumor penetration and off-target effects [8]. To overcome these limitations, the combination of BiTEs and OVs has been evaluated and displayed promising results [76,77]. Fajardo et al. designed and generated an oncolytic adenovirus armed with BiTE, known as ICOVIR-15K-cBITE. This new cBiTE-expressing adenovirus increased the accumulation and persistence of tumor-infiltrating T cells in human lung and colon cancer xenograft mouse models [76]. Wing et al. generated an oncolytic adenovirus armed with an epidermal growth factor receptor (EGFR)-targeting BiTE (OAd-BiTE) and demonstrated that OAd-BiTE with EGFR-targeting CAR-T therapy improved anti-tumor efficacy and prolonged survival in various mouse cancer models [77].

Recently, tri-specific killer cell engagers (TriKEs) have also been developed. Eric Vivier et al. generated a new trifunctional antibody that engages two activation receptors (NKp46 and CD16) on natural killer cells and a tumor antigen on cancer cells [78]. They demonstrated that this trifunctional antibody significantly decreased tumor size and improved survival in a Raji B lymphoma xenograft mouse model [79]. Overall, OVs can be used as genetic carriers for delivering BiKES or TriKEs to TME and thus need to be further verified through subsequent preclinical and clinical studies for cancer treatment.

## 5. Current Limitations of OVs for Cancer Treatment

Although OVs can induce anti-tumor immunity through multiple mechanisms and serve as an ideal platform for combination immunotherapy, there are still many issues to be solved to optimize OV-based immunotherapy. These include viral species, delivery platforms, intratumoral viral spread, and dosing strategies [5,8,27]. In addition, anti-viral immunity in the host immune system is continuously trying to clear OVs within the TME [80]. Here, we summarize the barriers of oncolytic virotherapy and discuss how to overcome these hurdles to translate OV more feasibly into clinical practice.

### 5.1. Choosing Optimal OV Species

A variety of viral species have been developed as OVs. Because different kinds of viruses have diverse sizes, shapes, genetic materials, and pathogenicity, understanding the unique biological characteristics of OVs is an essential step for developing the most effective anti-tumor oncolytic virotherapy [3,27]. For example, the size of the virus within the TME matters; smaller viruses infiltrate and spread more easily within tumors, while larger viruses have larger genomes allowing a greater number of therapeutic genes to be inserted [27]. Moreover, RNA viruses can replicate within the cytoplasm, but DNA viruses must enter the nuclei of the target cells to replicate. Thus, the tumor specificity of DNA viruses depends on interactions between nuclear transcription factors (NTFs) and viral promoter/enhancer elements, although RNA viruses are not under the control of NTFs [81]. These differences indicate that RNA viruses exert anti-tumor effects faster and are less selective with regards to tumors compared to DNA viruses. The presence of a viral capsid is also an important factor in OV development because enveloped viruses are less oncolytic and can be more easily cleared by the host immune system [27].

### 5.2. Efficient OV Delivery (Local or Systemic)

OVs can be delivered locally (mainly intratumorally) or systemically (mainly intravenously). Local intratumoral injection is the most common route of administration; intratumoral delivery maximizes the concentration of OVs at target tumor lesions while minimizing systemic toxicity [27,82,83]. However, this method cannot be applied to inaccessible or multifocal tumors. Furthermore, viable tumor cells at the OV injection site are essential for viral transfection and immune cell recruitment. In addition, treatment efficacy can vary depending on the skill level of the operator [83]. On the other hand, theoretically, systemic administration is an ideal delivery route because it is minimally invasive and highly repeatable, covering both primary and metastatic tumors. However, viral particles can be cleared rapidly by the host immune system, including neutralizing antibodies. To avoid this issue, envelope modification and the development of novel delivery systems using MDSCs as viral carriers have been explored as a means to deliver OVs to tumor sites [82].

New delivery platforms have also improved the therapeutic effects of OVs. Usually, viral particles are rapidly degraded by the host immune system. Various agents such as nanoparticles, liposomes, polyethylene glycol, and polymeric particles have been used to deliver OVs from the systemic circulation to the local TME [84,85]. Magnetic drug targeted systems have also become a promising carrier system to effectively deliver viruses to tumor cells [86]. Therefore, finding the optimal route of administration and enhancing the homing of OVs to tumor sites is pivotal for improving anti-tumor efficacy.

### 5.3. Enhancing Intratumoral OV Infiltration and Diffusion

The intratumoral distribution of OV within the TME is also a critical factor that determines its anti-tumor efficacy. Besides the aforementioned virus size and envelope type, a dense ECM within tumors serves as a physical barrier to intratumoral OV infiltration and diffusion [27,28,83]. To overcome this barrier, new OVs with specific enzymes capable of ECM degradation have been generated and have shown significant anti-tumor effects in preclinical studies [87,88]. For example, relaxin is a peptide hormone that can inhibit interstitial collagen synthesis and upregulate collagenase expression. Oncolytic adenoviruses expressing relaxin (YDC002) show potent anti-tumor effects in pancreatic tumor xenograft mice [87]. Moreover, Guedan et al. developed an oncolytic adenovirus (ICOVIR17), which expresses a soluble form of human sperm hyaluronidase (PH20). PH20 secreted from ICOVIR17 effectively degraded intratumoral hyaluronan, an important structural component of the ECM. Thus, ICOVIR17 showed enhanced intratumoral spread, better viral distribution, and more potent anti-tumor effect compared to the parental virus [89].

### 5.4. Anti-Viral Immunity

OV clearance through anti-viral immunity can also limit OV-induced anti-tumor efficacy [25,82]. There are many cases where anti-viral immunity already exists because most OVs used in anti-cancer therapy are human pathogens that are abundant in the environment. Moreover, repeated OV administration not only induces anti-tumor immunity but also triggers anti-viral immunity [82,90]. Anti-viral immunity can suppress viral replication, facilitate viral clearance, and attenuate anti-tumor activity in immunocompetent patients [25,82]. For example, neutralizing antibodies against Vaccinia virus target H3L envelope protein and interrupt viral-host fusion [91]. In the case of adenovirus, pre-existing neutralizing antibodies reduced the anti-tumor efficacy of oncolytic adenovirus [90]. Furthermore, T-VEC administration is limited to intratumoral injection because of high anti-HSV-1 antibody prevalence in humans [90,92]. In addition to pre-existing neutralizing antibodies, anti-viral immunity can also be mediated by the complement system, anti-viral cytokines, and non-specific uptake by off-target organs [82]. Therefore, to tackle anti-viral immunity, multiple strategies, including genetic manipulation of OV, cytokines, immunomodulators, nanoparticles, and the depletion of neutralizing antibodies, have been explored [82,83,90]. On the other hand, there are several conflicting reports suggesting anti-viral immunity could sometimes be beneficial for anti-tumor immunity because anti-viral immunity can recruit anti-tumor immune cells into the TME and reverse the immunosuppressive TME [5,27]. Thus, fine-tuning the balance between anti-viral and OV-induced anti-tumoral immunity may be important to maximize the efficacy of OV therapy.

### 5.5. Immunosuppressive TME

The immunosuppressive TME is another hurdle against effective oncolytic virotherapy. Tumor cells continuously secrete immunosuppressive chemokines and cytokines such as IL-10, TFG-β, and arginase-1 to reduce the amplitude of OV-induced anti-cancer immune responses [19,93,94]. To overcome the immunosuppressive TME, many OVs, including adenovirus, type I HSV, reovirus, and poxvirus have been genetically engineered to express GM-CSF to promote the maturation and differentiation of innate immune cells, thereby triggering immunostimulatory signals within the TME [24,27,83]. Moreover, researchers have modified OVs to express immune-activating cytokines (such as IL-2, IL-12, IL-18, IL-21, and IL-24) or chemokines (such as C-C motif chemokine ligand 5 (CCL5), CCL20, CCL21, and C-X-C motif chemokine ligand 10( CXCL10), thereby stimulating T cell proliferation, differentiation, and effector function within the TME [25,27]. Furthermore, some OVs have been engineered to express T cell costimulatory molecules such as OX40 (CD134), CD40, or 4-1BB to promote tumor-specific T cell activation and enhance anti-tumor immunity [22,79].

## 6. Conclusions and Perspective

Over the past two decades, OVs have emerged as promising immunotherapeutic agents against advanced cancers. Since the FDA approval of T-VEC as a new cancer therapy for patients with melanoma in 2015, many OVs have demonstrated modest anti-tumor efficacies with tolerable toxicity profiles as a monotherapy when administered through either intratumoral or systemic routes in clinical trials. To improve on this, researchers generated genetically modified OVs and combined them with other therapeutic modalities, most frequently with ICIs. In the future, researchers will develop new combination therapies with other agents, generate newly genetically engineered OVs, and produce new delivery systems. After overcoming current hurdles of oncolytic virotherapy, such as physical barriers, immunosuppressive TME, and host clearing of OVs, OVs will be the most powerful therapeutic strategy in cancer treatment.

## Figures and Tables

**Table 1:** Oncolytic viruses (OVs) under development (modified from Eissa et al. [33]).

Type of Virus	OV	Genetic Modifications	Cancer Type
Herpes simplex virus-1 (HSV-1, DNA virus)	T-VEC (talimogene laherparepvec, Imlygic)	ICP34.5 and ICP47 deletion, GM-CSF insertion	Melanoma [34]
	HF10 (canerpaturev, C-REV)	Loss of expression of UL43, Ul49.5, UL55, UL56, and LAT	Head and neck cancer [35]
	HSV1716 (Seprehvir)	ICP34.5 deletion	Extracranial cancers [36]
	G207	ICP34.5 deletion, ICP6 deletion, and LacZ insertion	Glioblastoma [37]
	G47∆	ICP34.5 deletion, ICP6 deletion, ICP47 deletion, and LacZ insertion	Breast cancer [38]
	OrienX010	ICP34.5 and ICP47 deletion, GM-CSF insertion	Melanoma [39]
Vaccinia viruses (DNA virus)	Pexastimogene devacirepvec (Pexa-Vec)	Thymidine kinase deletion, GM-CSF insertion	Hepatocellular carcinoma, renal cell carcinoma [5]
Adenoviruses (DNA virus)	H101 (Oncorine)	E1B deletion and E3 partial deletion	Head and neck cancer [39]
	ONYX-015	E1B-55 KDa gene deletion	Head and neck cancer [40]
	ONCOS-102 (formerly named CGTG-102)	adeno∆24-RGD-GM-CSF insertion	Mesothelioma [41] Prostate cancer [42] Melanoma [10] Ovarian and colorectal cancer [31]
	VCN-01	pRb-dependent; loaded with genes encoding PH20 hyaluronidase	Primitive neuroectodermal tumor [43]
	LOAd-703	pRb-dependent; loaded with genes encoding CD40L and 4-1BBL	Pancreatic cancer [44]
	DNX-2401	Deletion in 24bp in EIA and RGD-motif was engineered into the fiber H-loop, enabling the virus to use αvβ3 or αvβ5 an integrins to enter cells	Recurrent glioblastoma [45]
Reovirus (RNA virus)	Pelareorep (Reolysin)	Natural virus	Pancreatic cancer [46]
Paramyxoviridae (RNA virus)	Measles virus	hNIS insertion for MV-NIS and CEA insertion for MV-CEA	Multiple myeloma [47]
	Newcastle disease virus (NDV)	Natural virus	Cervical cancer [48]
Parvovirus (RNA virus)	Parvovirus H-1 (ParvOryx)	Natural virus	Neuroblastoma [49]
Picornaviruses (RNA virus)	CVA21 (Cavatak)	Natural virus	Melanoma, breast cancer [50]
	PVSRIPO	CD155/Necl5-dependent poliovirus; the internal ribosome entry site (IRES) of the poliovirus replaced with the IRES from human rhinovirus type 2 (HRV2)	Glioblastoma [51]

**Table 2:** Current clinical trials of combination therapy with OVs and immune checkpoint inhibitors (modified from Tao et al. [8]).

Type of Virus	OV	Immune Checkpoint Inhibitor	Phase	Cancer Type	Route of OV Administration	NCT Number
HSV-1	Talimogene laherparepvec, Imlygic (T-VEC)	Ipilimumab	I/II	Melanoma	IT	NCT01740297
		Pembrolizumab	III	Melanoma	IT	NCT02263508
		Pembrolizumab	I	Head and neck cancer	IT	NCT02626000
		Nivolumab	II	Lymphoma and non-melanoma skin cancers	IT	NCT02978625
	HF10 (canerpaturev, C-REV)	Ipilimumab	II	Melanoma	IT	NCT02272855
		Ipilimumab	II	Melanoma	IT	NCT03153085
Vaccinia virus	Pexastimogene devacirepvec (Pexa-Vec)	Ipilimumab	I	Advanced solid tumors	IT	NCT02977156
		Durvalumab/tremelimumab	I	Colorectal cancer	IV	NCT03206073
		Nivolumab	I/II	Hepatocellular carcinoma	IT	NCT03071094
		Cemiplimab	I	Renal cell carcinoma	IV, IT	NCT03294083
Adenovirus	ONCOS-102	Pembrolizumab	I	Advanced or unresectable melanoma	IT	NCT03003676
		Durvalumab	I/II	Advanced peritoneal cancers	IP	NCT02963831
	LOAd703	Atezolizumab	I/IIa	Pancreatic cancer	IT	NCT02705196
	p53 transduced adenovirus (Ad-p53)	Pembrolizumab	I/II	Head and neck cancer	IA	NCT02842125
		Nivolumab	II	Head and neck cancer	IT	NCT03544723
	Adenovirus vaccine expressing MAGE-A3 (Ad-MAGEA3)	Pembrolizumab	I/II	Non-small cell lung cancer	IM	NCT02879760
		Pembrolizumab	I	Metastatic melanoma and Squamous cell skin carcinoma	IM	NCT03773744
Coxsackie virus	CVA21 (Cavatak)	Pembrolizumab	I	Melanoma	IT	NCT02565992
		Pembrolizumab	I	Non-small cell lung cancer and bladder cancer	IV	NCT02043665
		Ipilimumab	I	Melanoma	IV	NCT03408587
Reovirus	Pelareorep (Reolysin)	Pembrolizumab	I	Advanced pancreatic adenocarcinoma	IV	NCT02620423
		Nivolumab	I	Relapsed/refractory multiple myeloma	IV	NCT03605719
VSV	VSV-hIFNbeta-sodium iodide	Avelumab	I	Malignant solid tumor	IT	NCT02923466
	VSV-IFNβ-NIS	Pembrolizumab	I	Non-small cell lung cancer and hepatocellular carcinoma	IV	NCT03647163
